# The BCL-2 Inhibitor Venetoclax Augments Immune Effector Function Mediated by Fas Ligand, TRAIL, and Perforin/Granzyme B, Resulting in Reduced Plasma Viremia and Decreased HIV Reservoir Size during Acute HIV Infection in a Humanized Mouse Model

**DOI:** 10.1128/jvi.01730-22

**Published:** 2022-11-30

**Authors:** Aswath P. Chandrasekar, Nathan W. Cummins, Sekar Natesampillai, Anisha Misra, Alecia Alto, Greg Laird, Andrew D. Badley

**Affiliations:** a Division of Infectious Diseases, Mayo Clinic, Rochester, Minnesota, USA; b Department of Molecular Medicine, Mayo Clinic, Rochester, Minnesota, USA; c Accelevir Diagnostics, Baltimore, Maryland, USA; Emory University

**Keywords:** BCL-2 family, venetoclax, human immunodeficiency virus

## Abstract

The BCL-2 prosurvival protein is implicated in HIV persistence and is a potential therapeutic target for HIV eradication efforts. We now know that cells harboring HIV are preferentially enriched for high BCL-2 expression, enabling their survival, and that the BCL-2 inhibitor venetoclax promotes the death of actively replicating HIV-infected cells *in vitro* and *ex vivo*. Herein, we assess the effect of venetoclax on immune clearance of infected cells and show that BCL-2 inhibition significantly enhances target cell killing induced by Fas ligand, TRAIL (tumor necrosis factor-related apoptosis-inducing ligand), and perforin/granzyme B and synergistically enhances autologous NK (natural killer) and CD8 cells’ killing of target cells. In a humanized mouse model of acute HIV infection, venetoclax monotherapy significantly decreases plasma viremia and normalizes CD4:CD8 ratios, and results in more mice with undetectable provirus levels than control. In this model, treatment was associated with leukopenia, as has been described clinically in patients receiving venetoclax for other indications. These data confirm meaningful anti-HIV effects of venetoclax during HIV infection but suggest that venetoclax use should be combined with ART (antiretroviral therapy) to reduce toxicity.

**IMPORTANCE** This study is the first to examine the applicability of BCL-2 inhibition in the setting of active HIV infection *in vivo*. Furthermore, this study demonstrates that venetoclax significantly enhances target cell killing induced by Fas ligand, TRAIL, and perforin/granzyme B and synergistically enhances autologous NK and CD8 cells’ killing of target cells.

## INTRODUCTION

Acute HIV infection quickly establishes an HIV reservoir consisting of integrated HIV proviruses, some of which enter a transcriptionally silent state, are long-lived, and persist even after antiretroviral therapy (ART) is initiated and maintained long term (1). A small subset of patients known as “elite controllers” have reduced viral reservoirs that are associated with enhanced anti-HIV immune function characterized by superior clearance of HIV-infected cells by CD8 and natural killer (NK) cells. These individuals harbor polyfunctional cytotoxic CD8 T-lymphocytes (CTL) that are capable of efficient degranulation of perforin/granzyme B, TNF-α, and other cytokines in response to HIV, express greater amounts of perforin, have more effective TRAIL (tumor necrosis factor-related apoptosis-inducing ligand) signaling (2) compared to patients who progress normally, and also demonstrate superior NK-cell-mediated HIV clearance compared to normal progressors. (3 to 9). In patients who cannot spontaneously control HIV replication, early initiation of ART leads to a smaller HIV reservoir size, which is associated with enhanced immunity and normalization of CD4:CD8 ratios (10).

Following exposure to cognate antigen, CD8 T cells activate, proliferate, and upregulate expression of death-inducing ligands Fas ligand, TNF (tumor necrosis factor), TRAIL, and the cytotoxic molecules perforin/granzyme B. Activated CD8 T cells then kill the antigen-expressing target cells through the nonexclusive pathways of perforin/granzyme B, FasL, TRAIL, and/or TNF. Importantly, each of these pathways have been implicated in the pathogenesis of CD4 depletion in HIV (11 to 13). NK cells do not recognize specific antigens but discriminate target cells from healthy cells through activating NK cell receptors such as NKG2D. HIV infection specifically upregulates ligands for this receptor, thereby enabling detection and elimination of HIV-infected cells. (14) Following target cell recognition, NK cells become activated and upregulate perforin/granzyme B as well as Fas ligand and TRAIL and use these pathways to kill target cells (15). Thus, TRAIL, FasL, and perforin and granzyme B are mechanisms commonly utilized by CTLs as well as NK cells to kill target cells, including HIV infected target cells (16 to 19).

During acute HIV infection, infected CD4 T cells die through a combination of immune cell killing of infected target cells, and the effects of viral replication that potentially occurs through multiple pathways, including autophagy, necroptosis, pyroptosis, or apoptosis (20 to 24). However, a subset of infected cells does not die, and persist long term either in the latent state or in an actively replicating state. This depends on host factors, such as the state of host immune control or the presence or absence of ART. (2, 25, 26).

The fact that a subset of HIV-infected cells does not die following acute HIV infection, and that most of those surviving cells are of the memory phenotype, led us to propose a selection hypothesis wherein the intrinsically long life of memory CD4 T cells could be linked, mechanistically, to their ability to survive HIV infection, facilitating HIV persistence. That hypothesis led to our understanding that memory CD4 T cells have higher BCL-2 expression and intrinsically resist death due to acute HIV infection (27, 28).

We and others have now shown that BCL-2^high^ CD4 T cells contain more HIV DNA than do BCL-2^low^ cells (29); that BCL-2^high^ CD4 cells in lymphoid follicles harbor the majority of HIV (30); and that BCL-2-overexpressing cells resist HIV protease-induced, Casp8p41-mediated, HIV-specific cell death (28). The combination of these findings has established BCL-2 as a critical regulator for the persistence of the HIV (20, 27). Subsequent studies verified that the inducible HIV reservoir was also disproportionately present in BCL-2^high^ subsets in *ex vivo* CD4^+^ T cells (29). Separately, the genetic overexpression of BCL-2 alone was found to be sufficient to recapitulate HIV latency, and this model system has been used to identify several clinically relevant approaches to latency reversal and elimination (31).

The signaling events downstream of TNF-α, FasL, and TRAIL binding their respective receptors (in the case of TRAIL, the death-inducing receptors TRAIL R1 and R2) involve caspase activation, which can be antagonized by overexpression of BCL-2 (32 to 34). Release of cytotoxic granules containing perforin and granzyme B leads to perforin oligomerization and pore formation in the outer target cell membrane, which allows granzyme to enter the cytoplasm of the target cell, where it cleaves and activates the BH3 interacting-domain death agonist (BID) to initiate apoptotic cell death (35), and this process is also antagonized by BCL-2 (20, 32, 36 to 38).

Because BCL-2 plays a critical role in the pathogenesis of human malignancy, selective BCL-2 inhibitors are now clinically approved for treating select human malignancies. The most notable of these is venetoclax (39), which has now become a widely prescribed and well-tolerated therapy for numerous hematological malignancies (40). In addition to selectively causing the death of malignant cells, such as chronic lymphocytic leukemia (CLL), recent reports suggest that venetoclax augments both CD8 and NK cell killing of malignant cells (41, 42). Given the promise of BCL-2 inhibitors in enhancing death of HIV infected cells, we assessed whether BCL-2 inhibition could enhance the ability of CD8 T cells and NK cells to kill acutely HIV-infected CD4 cells, and if those effects translated into meaningful changes in HIV dynamics and reservoir establishment in a humanized mouse model of acute HIV infection.

## RESULTS

### Venetoclax augments target cell killing by FasL, TRAIL, and perforin/granzyme B granule-mediated cytotoxicity against HIV-infected cells.

Understanding the pathways of target cell killing used by CTLs and that each of these effector pathways induces apoptotic cell death, we questioned whether venetoclax might synergize with Fas ligand, TRAIL, and perforin/granzyme B and therefore synergize with CTL effector function.

**FIG 1:**
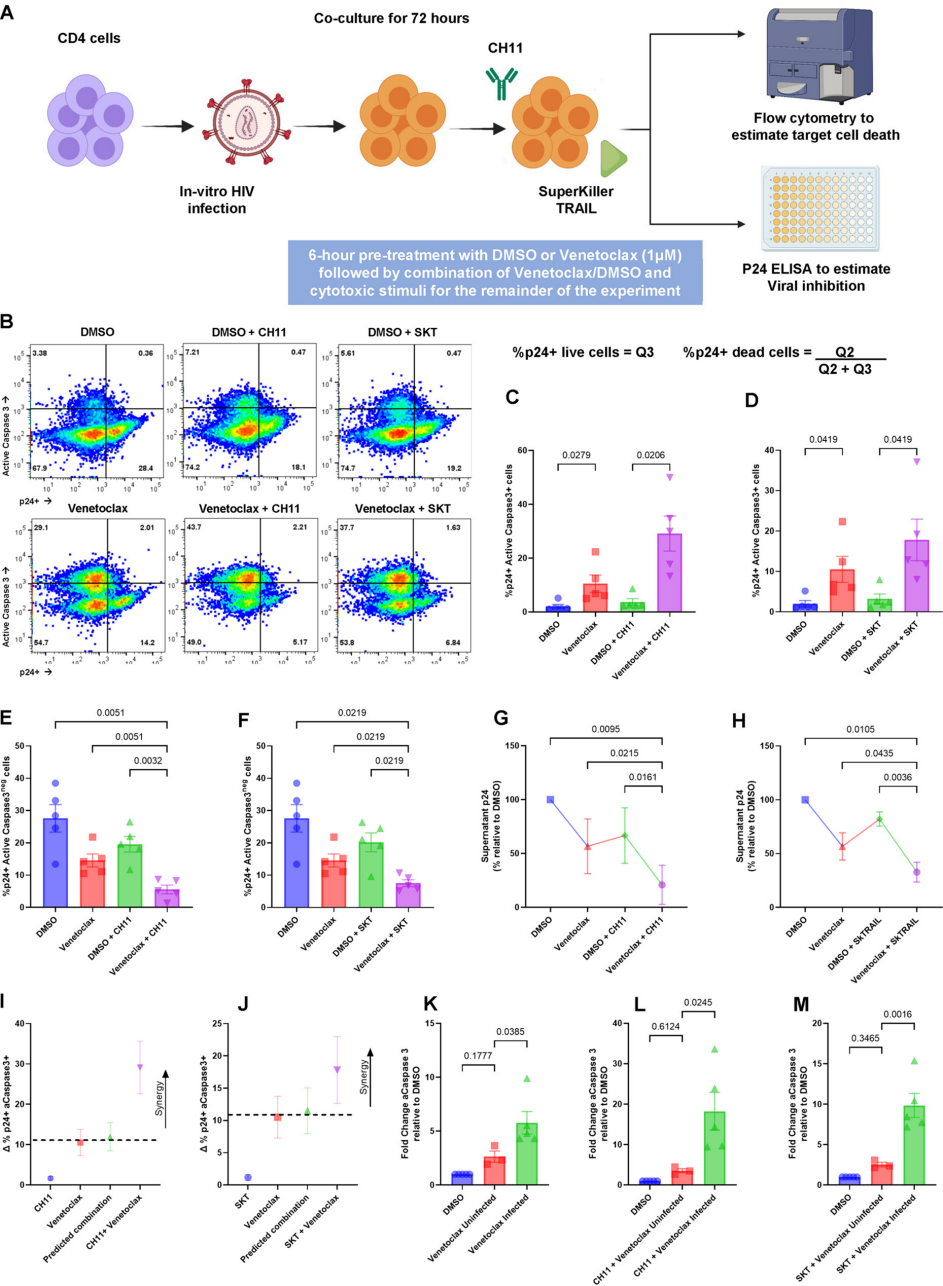
Venetoclax augments the HIV-infected cell clearance by FasL and TRAIL. CD4 T cells infected with HIV IIIb, *in vitro*, were treated with or without the BCL-2 inhibitor venetoclax (1 μM) or a vehicle control (DMSO) for 6 h followed by combination of DMSO/venetoclax with CH11-Fas-stimulating antibody (500 ng/mL), or SuperKiller TRAIL (SKT) (100 ng/mL) for 72 h. (A) Schematic of the experimental workflow for CH11 and SKT experiments. (B) Representative flow plots comparing the effect of DMSO and venetoclax on CH11 and SKT function. (C) Mean (SEM) of 5 experiments, demonstrating the proportion of p24-positive, caspase-3-positive cells with the addition of CH11 with or without venetoclax. (D) Mean (SEM) of 5 experiments, demonstrating the proportion of p24-positive, caspase-3-positive cells with the addition of SKT with or without venetoclax. (E) Mean (SEM) of 5 experiments, demonstrating the proportion of live p24-positive cells with the addition of CH11 with or without venetoclax. (F) Mean (SEM) of 5 experiments, demonstrating the proportion of live p24-positive cells with the addition of SKT with or without venetoclax. (G) Supernatant p24 values for the CH11 cultures (*n* = 4) relative to vehicle control. (H) Supernatant p24 values for the SKT cultures (*n* = 4). (I and J) Representations of Bliss independence calculations for CH11 and SKT, respectively; values greater than the predicted combination (dotted line) represent synergy. (K to M) Fold change in the percentage of aCaspase-3 positive cells, relative to DMSO, between inactivated, uninfected CD4 cells and p24-positive cells. Significance for the above plots calculated using a matched one-way ANOVA, with Holm-Sidak correction for multiple comparisons. Significance defined as *P* ≤ 0.050.

In our first assay, HIV IIIB-infected primary CD4 cells were treated with a static amount of CH11 (an agonistic anti-Fas antibody that mimics the effects of FasL), or SuperKiller TRAIL (SKT) (a preoligomerized recombinant TRAIL preparation that mimics the effects of TRAIL) in either the presence or absence of venetoclax (Fig. 1A).

The addition of venetoclax significantly increased the proportion of apoptosis induced by CH11 (a Fas agonistic antibody, *P = *0.0206) in the p24-positive CD4 cell population, as determined by active Caspase 3 staining, compared to control treatment (Fig. 1C). Similarly, we observed that apoptosis induced by SuperKiller TRAIL in the p24-positive CD4 cell population was also significantly increased by venetoclax compared to control (preoligomerized TRAIL, *P = *0.0419) (Fig. 1D). In this model system, where apoptosis-inducing ligands are added to cultures in bulk, we also observed increased apoptosis in the p24-negative target cell populations.

These treatments also reduced the proportion of p24-positive cells that remained after treatment (Fig. 1E and F), and correspondingly, CH11 and SKT treatments in the presence of venetoclax were accompanied by decreases in p24 detected in culture supernatant compared to CH11 and SKT alone. (Fig. 1G to H).

The ability of venetoclax in addition to CH11 or SKT to kill cells was synergistic, as determined by Bliss independence calculations (Fig. 1I and J).

It has previously been reported that HIV-infected cells express more death receptors (Fas receptor and TRAIL receptors 1 and 2) and are more susceptible to apoptosis induced by FasL and TRAIL than uninfected cells (11, 43 to 47). To assess in this model system, we repeated the experiment with unactivated, uninfected donor CD4 cells, versus HIV-infected cells, and compared cell death between infected and uninfected cells (relative to the dimethyl sulfoxide [DMSO] control), which revealed that HIV-infected p24-positive CD4s cells were significantly more susceptible to venetoclax (Fig. 1K) and that the combination of venetoclax and CH11 (Fig. 1L) and SKT (Fig. 1M). Venetoclax alone and in combination with CH11 and SKT did cause low-level death of uninfected, inactivated cells.

**FIG 2:**
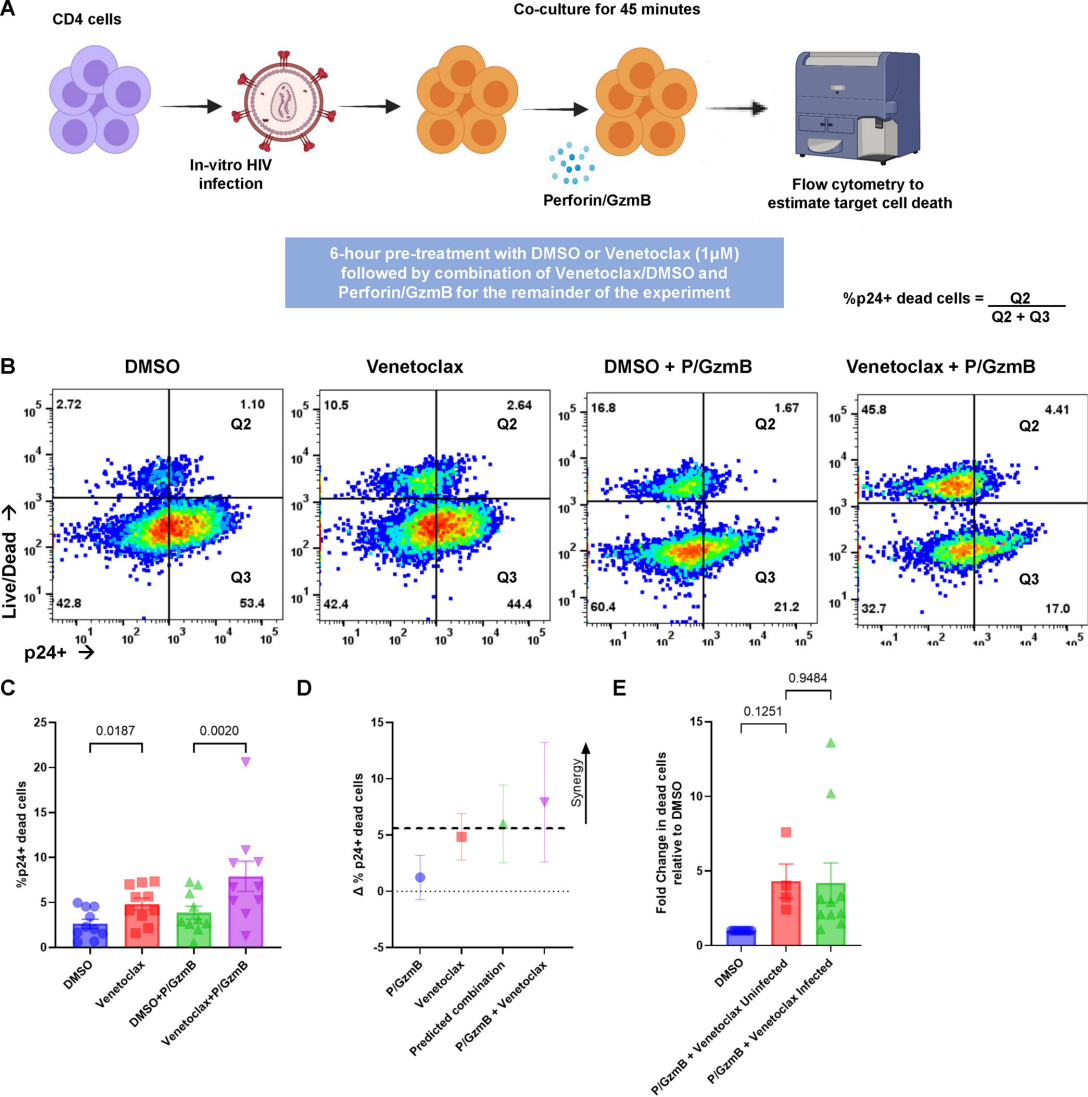
Venetoclax augments the HIV-infected cell clearance by perforin/granzyme B. CD4 T cells infected with HIV IIIb, *in vitro*, were treated with or without the BCL-2 inhibitor venetoclax (1 μM) or a vehicle control (DMSO) for 6 h, followed by a combination of DMSO/venetoclax with perforin/granzyme B for 45 min. (A) Schematic of the experimental workflow for perforin/granzyme B experiments. (B) Representative flow plots comparing the effect of vehicle and venetoclax on perforin/granzyme B function. (C) Mean (SEM) of 10 independent experiments demonstrating p24-positive target cell death following perforin/granzyme treatments, as determined by Live/Dead staining. (D) Representations of Bliss independence calculations for perforin/granzyme B. Values greater than the predicted combination (dotted line) represent synergy. (E) Fold change in the percentage of Live/Dead positive cells, relative to DMSO, between inactivated, uninfected CD4 cells and p24-positive cells. Significance for the above plots calculated using a matched one-way ANOVA, with Holm-Sidak correction for multiple comparisons. Significance defined as *P* ≤ 0.050.

In addition to inducing cell death through FasL and TRAIL, immune effector cells also kill target cells through perforin/granzyme B (48, 49), wherein perforin creates a pore in the outer membrane of target cells allowing granzyme B to enter, and granzyme B induces cell death in part through granzyme B cleavage and activation of Bid, which can be neutralized by BCL-2. (35) To experimentally assess whether venetoclax could also augment the activity of perforin/granzyme B, we treated infected cultures with recombinant perforin and granzyme B (Fig. 2A) and observed that venetoclax followed by perforin/granzyme B resulted in more death in the p24-positive cells than perforin granzyme B alone, as determined by live/dead staining compared to vehicle control and perforin/granzyme B (*P = *0.0090; Fig. 2C). Also, as this treatment was added to the bulk cultures, we also observed increased death of the P24-negative cells. The combination of perforin/granzyme B plus venetoclax had a mean Bliss independence coefficient of greater than 1, indicating synergy (Fig. 2D). In this model, where perforin/granzyme B is added to bulk cultures, similar amounts of cell death were observed in both HIV-infected and uninfected cells, relative to DMSO control (Fig. 2E).

Overall, our observations demonstrate that the BCL-2 inhibitor venetoclax sensitizes HIV-infected cells to death pathways used by CD8 T cells, and therefore suggests a mechanism to the prior observations. (29) Moreover, as NK cells use similar death pathways, our results suggest that venetoclax might also synergistically enhance NK-mediated killing of HIV-infected target CD4 T cells.

### Venetoclax enhances killing of productively HIV-infected CD4 T cells by both NK cells and CTL.

The above-described experiments demonstrate synergy between venetoclax and FasL, TRAIL, and perforin/granzyme B when these death-inducing proteins are supplied indiscriminately and to all cells in the cultures. This of course does not occur in patients, and instead these proteins are only delivered to cells following immune cell recognition (50 to 53), providing a measure of selectivity. CTL recognize cells through antigen presentation in the context of major histocompatibility complex (MHC) restriction. NK cells recognize infected target cells through upregulated NK triggering surface molecules such as NKp30, NKp44, and NKp46. (54, 55). Following their activation, NK cells increase expression of TRAIL and FasL and perforin/granzyme B (56 to 61). NK cells play an important role in control of HIV infection (62, 63), but alone fail to clear infection, so we sought to determine whether the addition of venetoclax could augment NK cell-effector function. This is of particular interest as venetoclax has recently been seen to augment the ability of NK cells to eliminate autologous acute myeloid leukemia (AML) cells (42).

**FIG 3:**
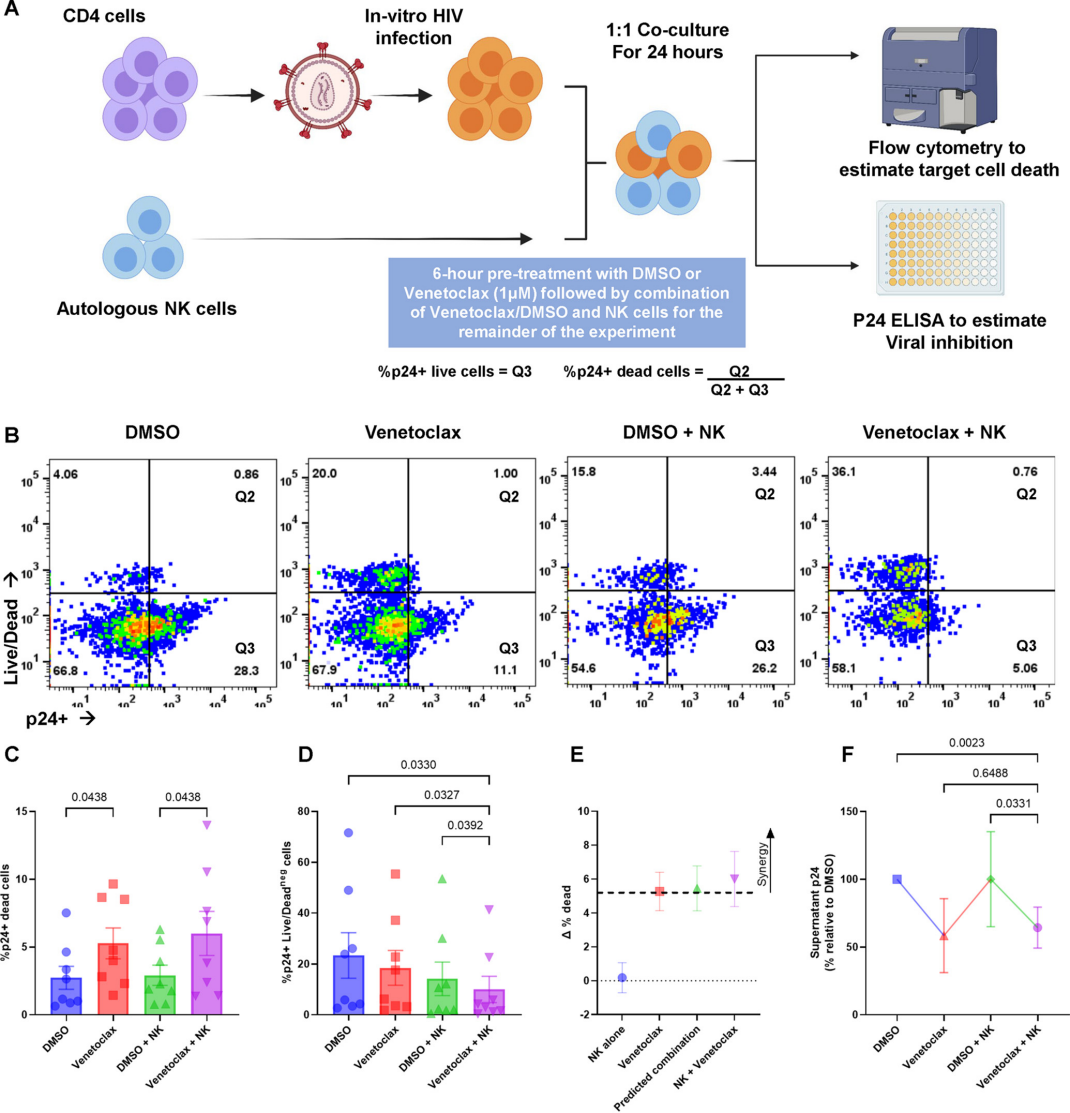
Venetoclax increases HIV-infected cell clearance by NK cells. CD4 T cells infected with HIV IIIb, *in vitro*, were treated with or without the BCL-2 inhibitor venetoclax (1 μM) or a vehicle control (DMSO) for 6 h followed by a combination of DMSO/venetoclax with autologous NK cells (1:1 E:T ratio) in duplicate, for 24 h. (A) Schematic of the experimental workflow for NK cell experiments. (B) Representative flow plots comparing the effect of vehicle and venetoclax on NK cell function. (C) Mean (SEM) of 8 experiments, demonstrating the proportion of p24-positive, target cells staining with Live/Dead stain. (D) Mean (SEM) of 8 experiments, demonstrating the proportion of live p24-positive cells in the NK cell cocultures. Significance for the above plots calculated using a matched one-way ANOVA, with Holm-Sidak correction for multiple comparisons. (E) Representations of Bliss independence calculations. Values greater than the predicted combination (dotted line) represent synergy. (F) Supernatant p24 values for the NK cocultures (*n* = 7) relative to vehicle control, significance calculated using a Friedman’s test, with Dunn’s test for multiple comparisons. Significance defined as *P* ≤ 0.050.

We cocultured primary human NK cells with autologous CD4 T cells that had been infected with HIV at 1:1 effector–target (E:T) ratios (Fig. 3A), and 24 h later cell death of p24-positive cells was determined. We observed increased cell death of p24-positive cells when incubated with NK cells plus venetoclax compared to NK cells plus control (*P = *0.0438) (Fig. 3C). At the time points assessed, the number of p24-positive cells was significantly decreased by treatments with NK cells and venetoclax, compared to NK cells alone (*P = *0.0392). (Fig. 3D), suggesting that the killing effect of the treatments had occurred at an earlier time. Bliss independence revealed synergy between NK killing and venetoclax, (Fig. 3E) and the addition of venetoclax to NK cells resulted in less supernatant p24 than with NK cells alone, further suggesting an anti-HIV effect. (*P = *0.0331) (Fig. 3F).

**FIG 4:**
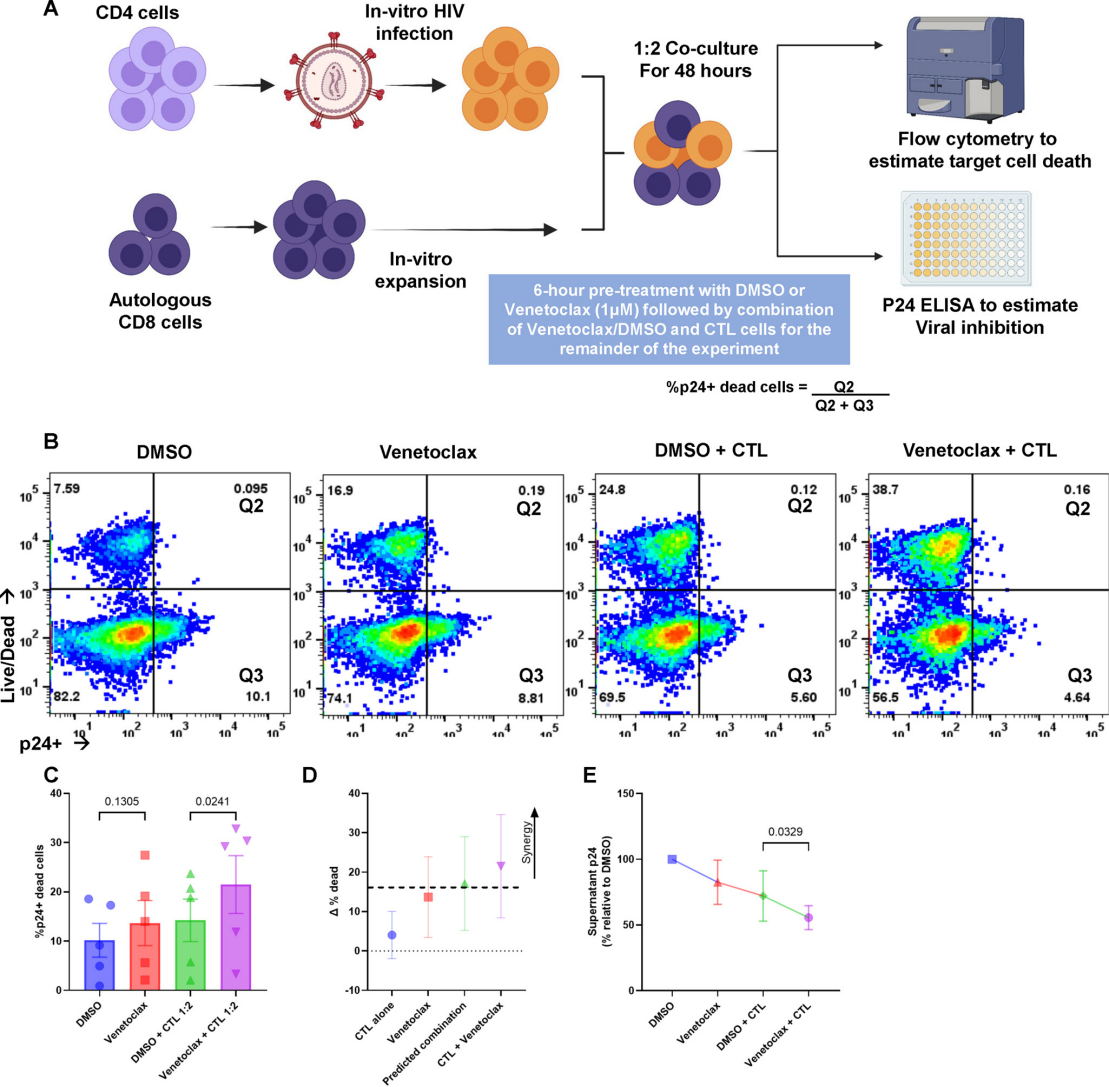
Venetoclax increases HIV infected cell clearance by cytotoxic T cells (CTLs). CD4 T cells infected with HIV IIIb, *in vitro*, were treated with or without the BCL-2 inhibitor venetoclax (1 μM) or a vehicle control (DMSO) for 6 h. The cells were subsequently cocultured with autologous, peptide-expanded CD8 (1:2 effector: target [E:T] ratio) for 48 h. (A) Schematic of the experimental workflow for CTL cell experiments. (B) Representative flow plots comparing the effect of vehicle and venetoclax on CTL function. (C) Mean (SEM) of 5 experiments, demonstrating the proportion of p24-positive target cells staining with Live/Dead stain. (D) Representations of Bliss independence calculations. Values greater than the predicted combination (dotted line) represent synergy. (E) Supernatant p24 values for the CTL cocultures (*n* = 5) relative to vehicle control. Significance for the above plots calculated using a matched one-way ANOVA, with Holm-Sidak correction for multiple comparisons. Significance defined as *P* ≤ 0.050.

CTL are also essential to control of HIV, yet that control alone is insufficient to clear HIV infection (64, 65). Venetoclax enhances CD8 T-cell killing of autologous HIV-infected CD4 T cells, including following reactivation from latency (29), and venetoclax also augments CD8 T-cell mediated clearance of cancer cells (41). Here, we tested the impact of the addition of venetoclax to CD8 cells and assessed the impact on HIV control in the setting of acute HIV infection. We used peptide-expanded CD8 cells cocultured with autologous HIV-superinfected CD4 T cells from ART-suppressed HIV-infected donors (Fig. 4A). Following 48 h coculture at 1:2 effector to target ratio, CTL plus venetoclax caused enhanced cell death in the p24-positive cells compared to CTL plus control (*P = *0.0241) (Fig. 4C). Bliss independence revealed that the difference between the observed and predicted change in p24-positive cell death was greater than 1, indicating synergy (Fig. 4D). Supernatant p24 levels were lower in cultures of CTL plus venetoclax-compared CTL-plus control, indicating that the anti-HIV effect of the combination was superior (*P = *0.0329) (Fig. 4E).

### BCL-2 inhibition during acute HIV infection of humanized mice reduces plasma viremia, decreases reservoir size, and normalizes CD4:CD8 ratios.

Despite the increasing evidence that venetoclax exerts anti-HIV effects, to date there are no studies that assess this putative effect *in vivo*. Moreover, there is concern about nonspecific toxicity of treatment with venetoclax toward uninfected CD4 T cells, as was seen in our *in vitro* experiments above. We therefore assessed whether, in a humanized mouse model of acute HIV infection, venetoclax alone has anti-HIV effect *in vivo*, and assessed the toxicity of such treatments *in vivo*.

NOD/Shi-scid/IL-2Rγnull (NSG) immune-deficient mice were engrafted with hematopoietic stem cells (CD34^+^ HSC) derived from 2 donors. The NSG-HSC mouse model is a well-established platform, widely used to investigate and validate the effects of anti-HIV therapies, possessing a fully differentiated human immune system that can support HIV replication and reservoir establishment, and reconstituting intact innate immunity capable of response (66, 67).

The mice were randomized into two groups of twenty-five mice each; one group was treated with vehicle and the other with venetoclax (100 mg/kg/day), as has been used clinically (68), administered daily by oral gavage over the 10-week experimental course, starting at day −1. On day 0, the mice were infected with 200 ng of HIV-1 (NL4-3 HIV1/Clade B; X4 tropism virus) by intraperitoneal injection. Clinical scores and body weight were monitored weekly, and blood collected for flow cytometry and PCR (Fig. 4A). At the time of sacrifice, we harvested mouse spleens for DNA isolation and estimation of HIV proviral load in tissue using the intact proviral DNA assay (IPDA).

Over the course of the 10-week experiment, we observed no significant changes in the body weights between treated mice and controls, and 16/25 mice survived to week 10 in the venetoclax-treated group versus 22/25 mice in the vehicle-treated group (*P* = NS).

**FIG 5:**
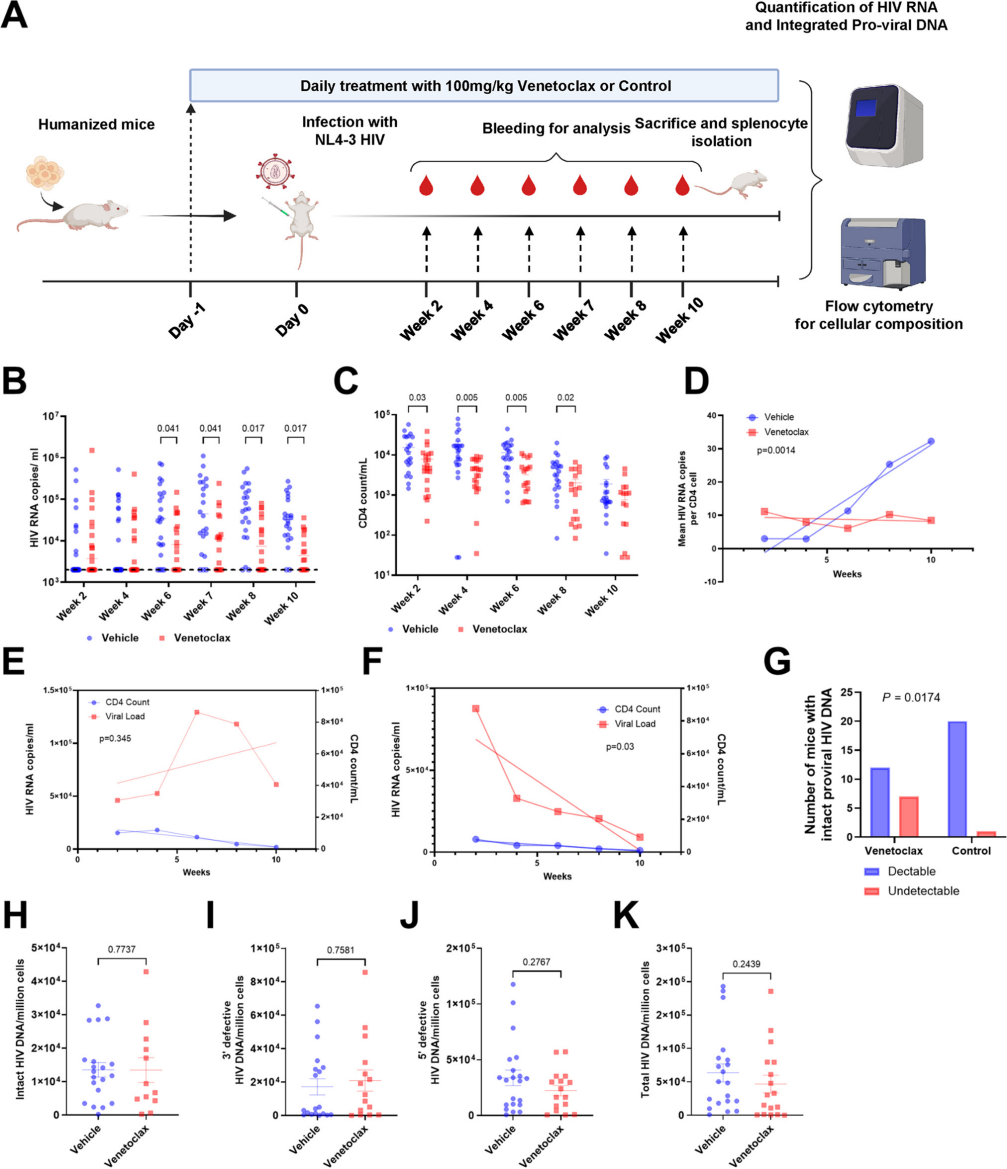
Venetoclax treatment reduces HIV load *in vivo*. Two groups of mice were treated with either venetoclax or vehicle control, with a total of 25 mice in each group, starting at day −1. The mice were infected with 200 ng of HIV-1 (NL4-3 HIV1/Clade B; X4 tropism virus) by intraperitoneal injection on day 0 and followed for 10 weeks. Blood obtained periodically was assayed for human T-cell counts and percentages by flow cytometry and HIV RNA by PCR, and splenocytes obtained after sacrifice were examined for intact proviral DNA by IPDA. (A) Schematic representing experimental workflow. (B) Mean (SEM) of plasma HIV RNA, expressed as copies per milliliter, over time. Significant reductions in plasma viral RNA were observed from week 6 onwards. Values reported as undetermined or less than the limit of detection were assigned the value 2,000 (the LOD for the assay, denoted by the dotted line). At 6 weeks, 20/23 control mice had HIV RNA above the LOD, compared to 12/18 venetoclax-treated mice; at 8 weeks, it was 20/22 (vehicle) compared to 11/18 (venetoclax); and at 10 weeks 20/22 (vehicle) compared to 10/16 (venetoclax). (C) Mean (SEM) of the CD4 count per mL over time. Significance calculated using multiple *t* tests with false-discovery rate (FDR) of <0.05. Significance defined as *P* ≤ 0.050. (D) Linear regression analysis comparing the mean number of viral copies per CD4 cell. (E) Linear regression analysis comparing plasma viremia and CD4 count in the vehicle-treated group. (F) Linear regression analysis comparing plasma viremia and CD4 count in the venetoclax-treated group demonstrating significantly greater rate of decline in viral load compared to CD4 count. (G) Seven of nineteen mice in the venetoclax group had undetectable intact proviral HIV DNA, compared to 1/22 in the vehicle-treated group. Significance calculated with Fisher’s exact test. (H to K) Comparison of intact, 3′-defective, 5′-defective, and total proviral DNA in the mice with detectable viral load as determined by IPDA.

We observed that HIV plasma viral load was significantly reduced in the venetoclax-treated mice by week 6 (*P = *0.041) and remained lower thereafter (Fig. 5B), compared to control mice. As expected in this acute HIV infection model, hCD4 cell number decreased in HIV-infected mice, and this decline was enhanced by venetoclax (Fig. 5C). To assess if venetoclax effects were a function of nonspecific killing of CD4 T cells, we assessed mean HIV plasma RNA, normalized to mean CD4 T-cell number. Plasma viremia normalized per CD4 T-cell number steadily increased over time in the HIV-infected and vehicle-control-treated mice. In contrast, in the HIV-infected and venetoclax-treated mice, plasma viremia normalized to CD4 T cell number stayed constant and was different than in the vehicle-control mice (*P = *0.0014) (Fig. 5D), suggesting that venetoclax had effects beyond nonspecific killing of CD4 T cells. That conclusion is further supported by linear regression analysis, which revealed that the rate of decline of HIV RNA copies per mL was different than the rate of decline in hCD4 number (*P = *0.03), which differed between venetoclax- and vehicle-control-treated mice. (Fig. 5 E and F).

We also estimated HIV reservoir size using IPDA, for intact provirus detection (69), to assess the number of copies of replication-competent provirus from tissue splenocytes harvested after sacrifice. Overall, we observed undetectable values of intact proviral HIV DNA in just one of the vehicle-treated mice (1/21), but in seven (7/19) mice that received at least 4 weeks of venetoclax therapy (*P = *0.0174) (Fig. 5G). In the subset of mice that had detectable provirus, the amounts of intact, 3′-defective, 5′-defective, and total proviral DNA were not different (Fig. 5H to K).

**FIG 6:**
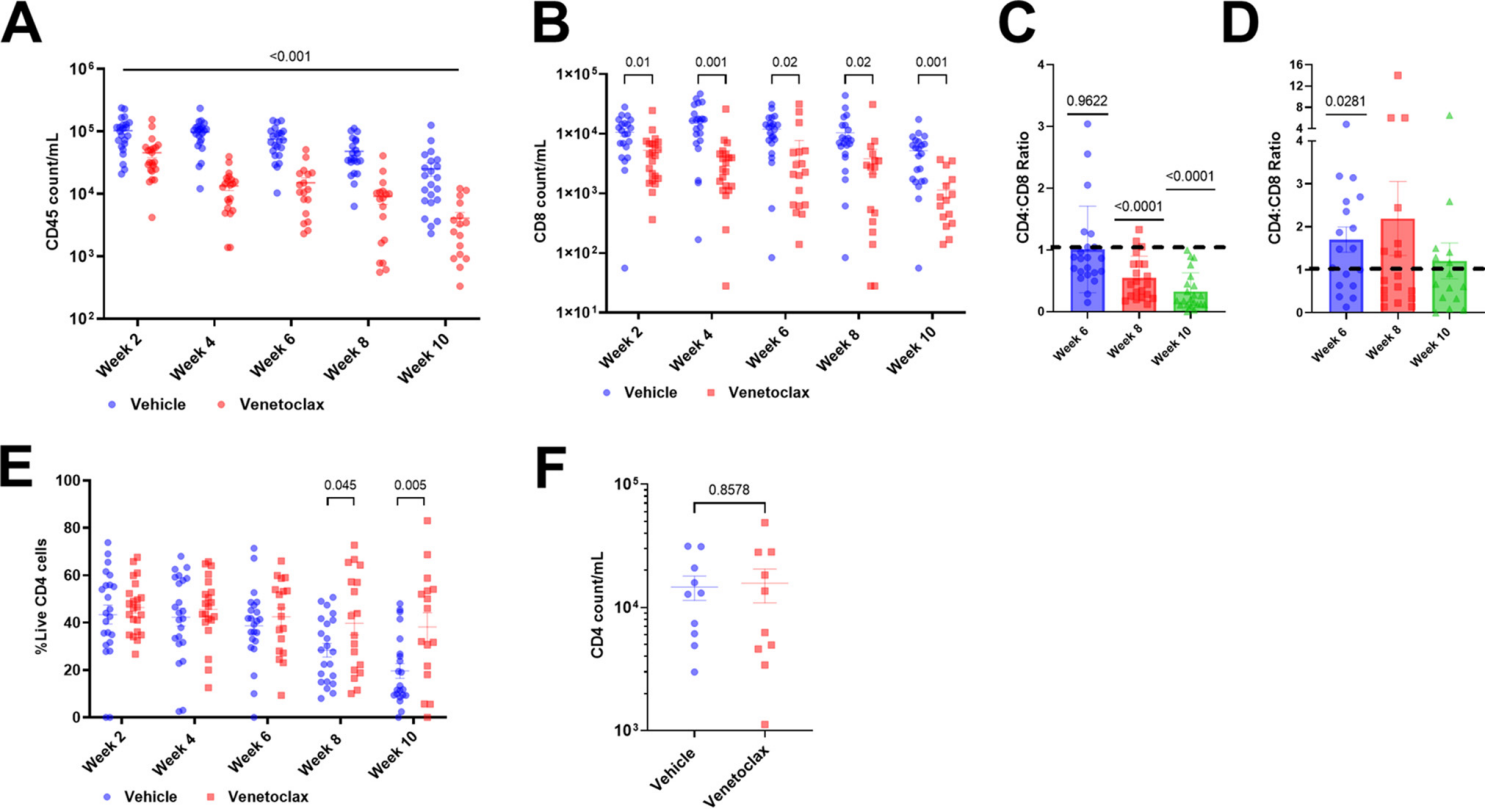
Cellular dynamics following venetoclax treatment *in vivo* in HIV infection. (A) Mean (SEM) of total CD45 cell count expressed as copies per milliliter, over time. (B) Mean (SEM) of the CD8 count per mL over time. (C and D) CD4:CD8 ratios in the vehicle- and venetoclax-treated groups, respectively. Significance calculated using a one-way *t* test compared to a hypothetical value of 1. (E) Percentage of live CD4 cells over time. (F) CD4 count at day 0 (*n* = 10) compared to vehicle (*n* = 10), calculated using an unpaired two-tailed *t* test.

Because mild leukopenia is observed clinically in patients receiving venetoclax for malignancy (70), we assessed cellular dynamics of treated mice; hCD45- and hCD8-positive cells declined in the venetoclax-treated group compared to control (Fig. 6A and B), yet CD4:CD8 ratios normalized to >1 at week 6 in the venetoclax-treated group (*P = *0.0281) (Fig. 6D), and we observed an increased percentage of hCD4 cells at weeks 8 and 10 (*P = *0.045 and 0.005) (Fig. 6E). In those mice for which we obtained cell counts after 24 h of venetoclax (week 0, *n* = 10, and *P* = NS), but before HIV infection, no differences in CD4 cell number were observed (Fig. 6F).

## DISCUSSION

Understanding cellular and molecular mechanisms within HIV-infected cells that impact their propensity for survival versus cell death is critical to identify novel therapeutic targets within infected cells that will increase their sensitivity to cell death, to inform efforts to develop more effective interventions for an HIV cure. The cumulative data with BCL-2 antagonists (28, 29, 71 to 73), coupled with our current data, argue that venetoclax, as a monotherapy, can achieve significant HIV clearance.

To date, we and others have established a role for BCL-2 as a critical determinant of HIV-infected cell survival in *ex vivo* experiments of acute HIV infection, in maintenance of the HIV reservoir, and following reactivation from latency (28, 29, 65, 71, 72). Herein, we establish the first proof of concept that *in vivo*, BCL-2 function is a critical determinant of HIV viral dynamics and HIV reservoir establishment, since BCL-2 antagonists result in reduced HIV viremia and more infected animals having an undetectable HIV reservoir (as assessed by IPDA). Moreover, we establish that BCL-2 inhibitors are synergistic with both of the death-inducing ligands Fas Ligand and TRAIL, as well as increasing perforin/granzyme B function, thereby providing a mechanistic basis to combine strategies that augment NK or CTL effector function with BCL-2 antagonists to promote death of HIV-infected cells. Observations here are in alignment with a previously published report that venetoclax augments immune effector cell function against HIV-infected targets, mediated by CTLs (29). Furthermore, we have extended these observations to HIV clearance mediated by NK cells through enhanced proapoptotic signaling through the immune effector pathways of FasL, TRAIL, and perforin/granzyme B. It is also noteworthy that CAR-T cells use killing pathways like those of native T cells, and thus in cancer model systems CAR-T cell killing of target cells is enhanced by venetoclax (74).

This newly demonstrated synergy between BCL-2 antagonists and immune-effector functions provides a rationale for testing venetoclax with immune-boosting agents to assess additive or synergistic effects on reducing HIV reservoir size. Already, groups are assessing the combinations of venetoclax and latency reversal agents using *in vitro* studies of HIV. (27, 75).

In the current era, there are numerous antiviral agents that successfully target HIV replication and can control HIV replication in infected individuals; therefore, although venetoclax also exerts antiviral effects, we do not propose using venetoclax during uncontrolled viral replication, as this is associated with significant toxicity in our mouse experiments. The toxicity of venetoclax in CLL is proportional to disease burden; in CLL patients with high tumor burden, administration of venetoclax causes tumor lysis syndrome; however, in patients with reduced tumor burden, toxicity is much less, and venetoclax is very well tolerated (76). We suggest a similar approach in the setting of HIV: the use of venetoclax only in settings where HIV replication is low, such as in ART-suppressed individuals, and to test the combinations of venetoclax with latency reversal agents, and possible immune enhancing interventions, to augment the likelihood of causing the death of individual cells that reverse from latency, while minimizing the potential for toxicity.

Our findings establish that venetoclax functions as a host-directed therapy that augments the death of cells containing replicating HIV during acute infection and that augments HIV clearance through both NK and CD8 T cells, through both death-inducing ligand and granule-mediated exocytosis pathways. Having established these results *in vitro* and as demonstrated here *in vivo*, we conclude that venetoclax alone meaningfully impacts HIV viremia and proviral load *in vivo*, providing a proof of concept for BCL-2 inhibition as an antiviral therapy, in an *in vivo* setting. These findings establish a significant role for BCL-2 as a determinant of viral dynamics in HIV infection and suggest that further investigation of BCL-2 inhibition for HIV cure efforts is warranted in the setting of ART suppression along with other cure-directed interventions.

## MATERIALS AND METHODS

### Humanized mouse model of HIV infection.

Before HIV infection, the humanized mice were randomized into 2 groups, with 25 mice per group, according to their hCD4+ T-cell levels, humanization rate, and cord blood donor such that the range of values were balanced between groups for each of these categories. Treatments with either vehicle or venetoclax (100 mg/kg) were initiated at day −1 and administered daily by oral gavage over the 10-week experimental course. On day 0, mice were infected with 200 ng of HIV-1 (NL4-3 HIV1/Clade B; X4 tropism virus) by intraperitoneal injection. Clinical scores and body weight were monitored weekly.

Blood (100 μL) was collected every 2 weeks from the retro-orbital sinus into EDTA tubes. The plasma was separated, heat-inactivated, then frozen at −80°C. RNA was extracted from 40 μL of inactivated plasma using an automated nucleic acid purification device (Arrow, NorDiag) and Viral RNA Extraction kit (DiaSorin, 12-08-02). HIV plasma viral load was determined with qRT-PCR and Generic HIV Charge Virale (Biocentric, TR001-440IC). The limit of sensitivity was 2,000 copies/mL. Undetermined values and values reported below 2000, were assigned the limit of detection (LOD) value of 2,000.

Flow cytometry analysis was performed on an Attune NxT Flow Cytometer (Life Technologies), using fresh blood incubated with Human FcR blocking reagent and stained with the anti-human antibodies anti-CD3-BV421 (Biolegend), anti-CD8-PerCP/Cy5.5 (Biolegend), anti-CD45-BV510 (Becton, Dickinson), and anti-CD4-BV711 (Becton, Dickinson), as well as with viability dye (Fixable Yellow LIVE/DEAD, Thermo Fischer), for 30 min at 4°C. Red blood cells were lysed with RBC lysis buffer, and cells were fixed by using a BD Cytofix/Cytoperm kit.

Mouse spleens were collected at sacrifice (day 70), and DNA was isolated from dissociated cells (TransCure bioServices). The DNA was analyzed by Accelevir Diagnostics for IPDA, as previously described (69). Briefly, primers targeting both the 5′ψ sequence and the 3′*env* sequence of HIV were amplified using digital droplet PCR to differentiate intact sequences of proviral DNA.

### Human cell isolation and culture materials.

Primary peripheral blood mononuclear cells (PBMCs) from blood bank donors, and PBMCs from virologically suppressed HIV-positive subjects, were obtained through 2 Mayo Clinic-approved institutional review board (IRB) protocols (13-005646 and 16–001938). CD8 and CD4 T cells and NK cells were isolated using RosetteSep or EasySep Negative selection kits (Stem Cell Technologies, Vancouver, Canada). Cells were cultured in RPMI 1640 medium with 10% fetal bovine serum (FBS), 2 mM l-glutamine, 100 units/mL penicillin, and 100 μg/mL streptomycin (complete RPMI).

### Death-inducing ligand experiments.

CD4 T cells were activated for 48 h with 20 ng/mL phytohemagglutinin (PHA) and 50 U/mL IL-2, washed once with RPMI, and infected with HIV IIIB (NIH AIDS reagent) as previously described (77). At 48 h after infection, 1 × 10^6^ CD4 T cells/mL were cultured for 6 h with DMSO (vehicle control) or 1 μM venetoclax, followed by a combination of DMSO or venetoclax (1uM) with CH11-Fas-stimulating antibody (500 ng/mL), or SuperKiller TRAIL (100 ng/mL). After 72 h, cells were fixed with 2% paraformaldehyde (PFA), permeabilized with 0.1% NP40, and stained intracellularly with a p24-PE mouse monoclonal antibody (Clone KC57, Beckman Coulter) and FITC-conjugated caspase-3 antibody. The culture supernatants were stored at −80°C. Culture supernatant was analyzed for p24 viral protein using the ZeptoMetrix HIV Type 1 p24 Antigen ELISA kit (catalog number 0801111), as per the manufacturer’s protocol.

### Perforin/granzyme experiments.

Briefly, CD4 T cells were isolated and activated for 48 h with 20 ng/mL PHA and 50 U/mL IL-2 and infected with HIV IIIB as above. At 48 h after infections, the CD4 T cells were cultured for 6 h with DMSO (vehicle control) or 1 μM venetoclax, then resuspended in 30 μL of “Buffer C (cell suspension)” as described in Thiery et al. (78) (formulated as Hanks' balanced salt solution [HBSS]; 10 mM HEPES; 4 mM CaCl_2_; 0.4% [wt/vol] bovine serum albumin).

Super mixes of perforin/granzyme B were prepared in 30 μL “Buffer P (peptide)” (formulated as HBSS, 10 mM; HEPES). The supermix containing perforin/granzyme B was added to the cell suspension, in combination with DMSO/venetoclax, and the culture was carried out for 45 min. The cells were harvested and stained with Live/Dead FITC fixable stain (Invitrogen), fixed overnight with 2% PFA, then permeabilized with 0.1% NP40 and stained with the p24-PE antibody. Cells were gated on perpendicular axes of Live/Dead and p24. Cells that were double positive were divided by the number of live p24 cells to calculate the percentage of p24-positive cells that were also Live/Dead positive.

### NK cell coculture experiments.

CD4 T cells and NK cells from the same donor were isolated. CD4 T cells were activated for 48 h with 20 ng/mL PHA and 50 U/mL IL-2, and infected with HIV IIIB as described above. NK cells were maintained in RPMI + 50 U/mL IL-2 at 37°C. Forty-eight hours following infection, the CD4 T cells were washed and resuspended in fresh media at a concentration of 1 × 10^6^ cells/mL with DMSO (vehicle control) or 1 μM venetoclax, and cocultures with autologous NK cells were established at a 1:1 E:T ratio. Cells were collected after 24 h of coculture, stained with Live/Dead FITC fixable stain (Invitrogen) and CD56-APC (Invitrogen), and fixed, and the culture supernatants were stored at −80°C. The cells were permeabilized with 0.1% NP40 and stained with a p24-PE antibody. Cultures were analyzed with flow cytometry by gating on CD56-negative cells against a forward scatter axis (Fig. S1C, panel 4). These were designated target cells and were gated on perpendicular axes of Live/Dead and p24. All cells staining positive for Live/Dead were used for subsequent statistical analyses, using the average readings for duplicate wells. Culture supernatants were analyzed for p24 viral protein using the ZeptoMetrix HIV Type 1 p24 Antigen ELISA kit, as above.

### HIV CTL generation.

HIV-specific CTL were enriched from the PBMCs of ART-suppressed HIV-infected individuals by pulsing with Gag, Pol, and Nef Pepmixes in the presence of IL-7 (final concentration 10 ng/mL) and IL-15 (final concentration 5 ng/mL). The CTL were cultured on a G-Rex10 plate (Wilson Wolf Manufacturing, 80040S) for 12 to 15 days as previously described (79).

### CTL coculture experiments.

Cryopreserved PBMCs from HIV-positive, ART-suppressed individuals were used to isolate CD4 T cells and CD8 T cells. CD8 T cells were enriched for HIV-specific CTL as above, and CD4 T cells were HIV-infected as described above.

On the day of coculture, viable cells were counted and resuspended in fresh RPMI and 10 U/mL IL-2. The CD4 cells were divided into 2 treatment groups and received DMSO or venetoclax (1 μM) pretreatment for 6 h. CD8 cells were counted, and the coculture was carried out at E:T ratios of 1:2, in combination with DMSO/venetoclax.

At 48 h, the cells were collected for analysis via flow cytometry, and the culture supernatants were harvested. The cells were stained with Pacific Blue Mouse Anti-Human CD8 (catalog number 558207) and LIVE/DEAD Fixable Green Dead Cell Stain (catalog number L23101), fixed with 2% PFA, then permeabilized with 0.1% NP40 and stained with the p24-PE mouse monoclonal antibody. Briefly, CD8-negative cells were selected for, gating against a forward scatter axis (Fig. S1D, panel 4). These were designated target cells and were gated on perpendicular axes of Live/Dead and p24. All cells staining positive for Live/Dead were used for subsequent downstream statistical analyses. Culture supernatants were analyzed for p24 viral protein using the ZeptoMetrix HIV Type 1 p24 Antigen ELISA kit.

### Statistical methods.

Graphs were made by using GraphPad Prism 9 (GraphPad, Inc.) and show means ± the standard error of the mean, unless otherwise stated. Statistical analyses were performed using GraphPad Prism, and the types of tests used for analysis are listed in the corresponding figure legends. *P* values of ≤0.050 were considered significant. Data sets were assessed for normal or logarithmic distribution through Shapiro-Wilk testing in GraphPad Prism, and parametric or the equivalent nonparametric tests were subsequently chosen. Bliss independence values were calculated as previously described, and a difference in mean of >1 from the predicted value was considered synergy (80). Repeated-measures ANOVAs were adjusted for multiple comparisons using the Holm-Sidak test, comparing the specific groups of interest illustrated in the figures. Multiple *t* tests and multiple Mann-Whitney tests used in the mouse experiments were adjusted using two-stage step-up method of Benjamini, Krieger, and Yekutieli with a false discovery rate threshold of 0.05 (81).

### Study approval.

All procedures described in this study involving mice have been reviewed and approved by the local ethics committee (CELEAG). Primary peripheral blood mononuclear cells (PBMCs) from blood bank donors and PBMCs from virologically suppressed HIV-positive subjects were obtained through 2 Mayo Clinic-approved IRB protocols (13-005646 and 16–001938).
