# Supplementary material for: The BCL-2 Inhibitor Venetoclax Augments Immune Effector Function Mediated by Fas Ligand, TRAIL, and Perforin/Granzyme B, Resulting in Reduced Plasma Viremia and Decreased HIV Reservoir Size during Acute HIV Infection in a Humanized Mouse Model
Source: J Virol. 2022 Nov 30;96(24):e01730-22. doi: 10.1128/jvi.01730-22 (PMC9769373; doi:10.1128/jvi.01730-22)
Supplement: Supplemental file 1 — Fig. S1. Download jvi.01730-22-s0001.pdf, PDF file, 0.4 MB [file jvi.01730-22-s0001.pdf]

Supplemental Figure 1

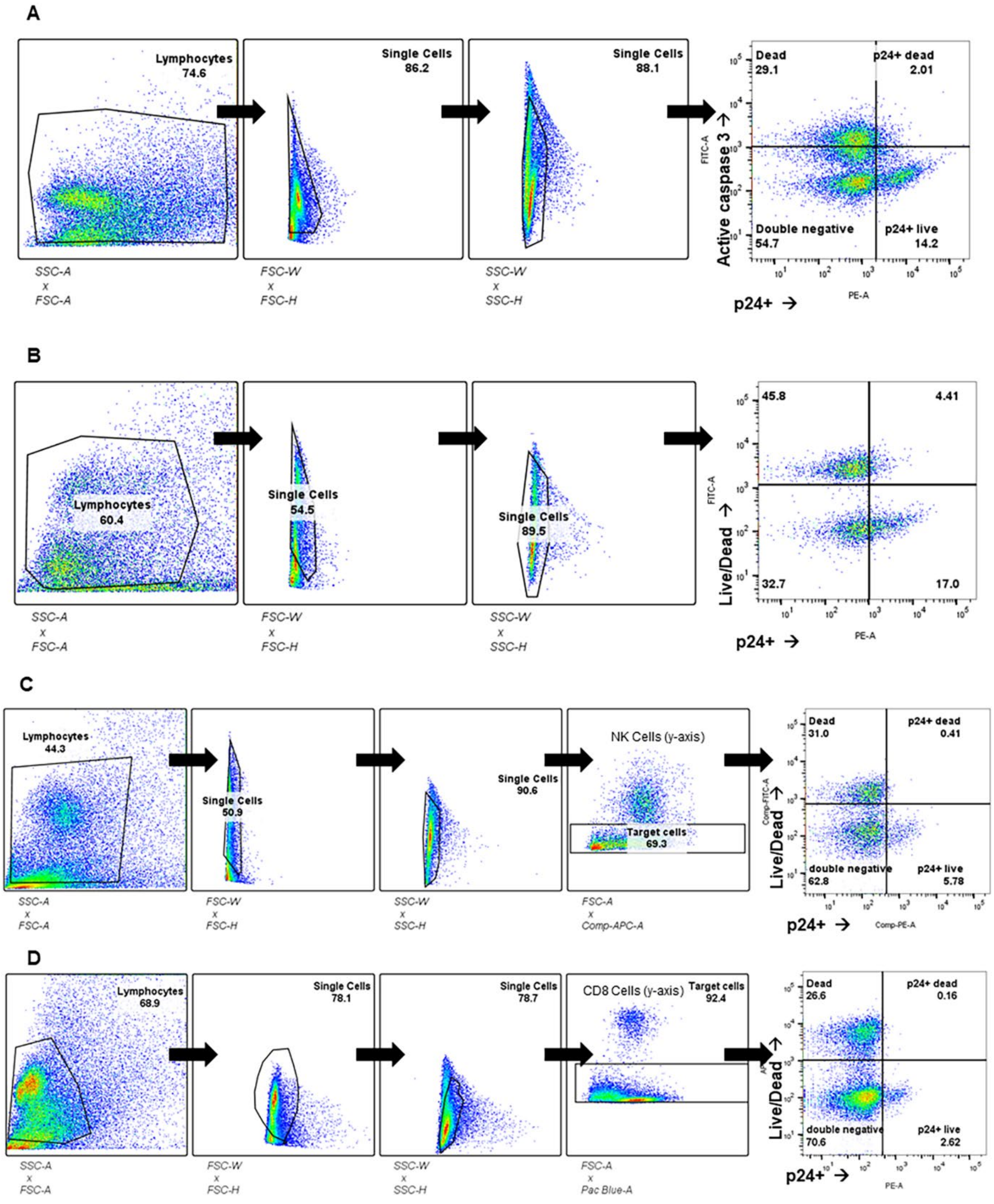

Supplemental figure 1: Gating strategy for figures 1-4

A Gating strategy for CH11 and SKT experiments. **B** Gating strategy for Perforin/Granzyme B experiments. **C** Gating strategy for NK experiments. NK cells were stained with an APC conjugated antibody and excluded from the target cell population (panel 4). **D** Gating strategy for CD8 experiments. CD8 cells were stained with a PacBlue conjugated antibody and excluded from the target cell population (panel 4).
